# Retrospective analysis of omega-3 fatty acids and the DASH diet in hyperlipidemia and hypertension management among obese individuals

**DOI:** 10.3389/fnut.2025.1704552

**Published:** 2025-11-21

**Authors:** Hongtao Yin, Yanqing Zhou, Yingjun Zhou, Lin Ren, Lixiang Ma

**Affiliations:** 1 Department of Cardiology, The First Hospital of Qinhuangdao, Qinhuangdao, Hebei, China; 2 Department of Operating Room, The First Hospital of Qinhuangdao, Qinhuangdao, Hebei, China

**Keywords:** obesity, DASH diet, omega-3 fatty acids, hyperlipidemia, hypertension, cardiometabolic health

## Abstract

**Background:**

Cardiovascular disease, obesity, high blood pressure, and abnormal lipid profiles are public health issues. Dietary therapies like the DASH diet and omega-3 supplements show cardiometabolic advantages in Western populations. Few Asian cohort studies have examined the effects on obese people with dyslipidemia and hypertension.

**Objective:**

The study aims to evaluate the associations of omega-3 supplementation and DASH diet adherence, individually and in combination on lipid profiles, blood pressure, and metabolic outcomes among Chinese obese adults.

**Methods:**

This retrospective analysis examined data from 3,350 obese adults (BMI ≥ 28 kg/m^2^) with hyperlipidemia and hypertension, recruited at First Hospital of Qinhuangdao City from March 2023 to February 2025. Validated food frequency surveys and structured self-reports examined dietary adherence, while prescriptions and self-reports confirmed omega-3 consumption. LDL-C, HDL-C, triglycerides, total cholesterol, and systolic and diastolic blood pressure were the main results. Weight loss, glycemic control, and lipid/blood pressure targets were secondary outcomes. Multivariable linear, logistic, and mixed-effects regression models were used for statistical analysis.

**Results:**

Among participants, 42.7% used omega-3 supplements, 35.2% adhered to the DASH diet, and 22.1% followed both interventions. Combined adherence was associated with the greatest improvements: LDL-C reduction (−31.7 mg/dL), HDL-C increase (+5.8 mg/dL), triglyceride reduction (−45.3 mg/dL), and systolic/diastolic BP reduction (−14.7 mmHg, all *p* < 0.001). Participants with combined adherence were significantly more likely to achieve both lipid and BP targets (68.6%; OR = 3.74, 95% CI: 3.20–4.40) than those with single interventions. Subgroup analyses revealed stronger benefits among older adults, women, individuals with longer obesity duration, and those with diabetes. Time-dependent analyses confirmed sustained improvements over 24 months. Adverse events were generally mild, with overall adherence remaining above 80%.

**Conclusion:**

Combined omega-3 supplementation and DASH diet adherence was associated with synergistic improvements in lipid and blood pressure control, weight reduction, and glycemic outcomes. These findings support the implementation of integrated dietary and supplement-based strategies for cardiometabolic risk reduction in obese adults, particularly in Asian populations.

## 1 Introduction

Obesity is a chronic and multifactorial disorder characterized by excessive adiposity, metabolic dysregulation, and low-grade systemic inflammation. It is strongly associated with the development of hyperlipidemia, hypertension, and cardiovascular disease (1, 2). Since 1975, obesity has nearly tripled, leaving over 650 million individuals obese worldwide. Over the previous few decades, China’s obese population has grown. About 16–18% of US individuals are obese, while over 50% are overweight or obese. Along with this epidemiological shift, obesity-related comorbidities have increased significantly. About 35–40% of Chinese individuals have hyperlipidemia, and nearly 27.5%, or 245 million people, have hypertension. Since obesity, dyslipidemia, and hypertension cause cardiovascular morbidity and mortality, this disrupts public health (3, 4). The metabolic, hormonal, and circulatory pathways linking obesity, dyslipidemia, and hypertension are complex. Adipose tissue liberates fatty acids. Triglycerides and VLDL are overproduced by the liver. The liver requires time to balance them, so the cycle continues. Reduced lipoprotein lipase activity and hypertriglyceridemia result from obesity-induced insulin resistance (5). Obesity increases blood pressure through renin–angiotensin–aldosterone system activation, sympathetic nervous system overactivity, endothelial dysfunction, and vascular remodeling. Additionally, adipose-derived cytokines like TNF-*α* and interleukin-6 cause vascular stiffness and inflammation, worsening hyperlipidemia and hypertension in obese persons (6).

Nutritional adjustments are needed for non-pharmacological cardiometabolic disease treatment. Numerous diets lower blood pressure and enhance lipids, including DASH. The DASH diet limits salt, saturated fat, and added sugar and promotes fruits, vegetables, whole grains, legumes, nuts, and low-fat dairy. High fiber and antioxidant intakes improve insulin sensitivity, reduce oxidative stress, and change lipid metabolism, while potassium, magnesium, and calcium increase vasodilation and sodium excretion (7, 8). Several meta-analyses showed that the DASH diet improves cardiovascular health and cholesterol markers by lowering lipids and improving blood vessel function. Inhibiting diacylglycerol acyltransferase decreases liver triglyceride production. They also increase lipoprotein lipase activity. The ultimate benefit is improved *β*-oxidation of fatty acids (9). In addition, omega-3 fatty acids compete with arachidonic acid in eicosanoid pathways, creating resolvins and protectins. These molecules are formed by anti-inflammatory and vasodilatory mediators. All of these effects lower plasma lipids, vascular inflammation, endothelial nitric oxide bioavailability, and arterial stiffness. Clinical research indicated that high dosages of eicosapentaenoic acid reduced triglycerides by 20–30% and serious adverse cardiovascular events by 25% (10).

Omega-3 fatty acids and the DASH diet alleviate metabolic problems that cause high blood pressure and cholesterol. Nutrients and antioxidants lower blood pressure and cholesterol in the DASH diet. However, omega-3 s greatly lower triglycerides and inflammation. Combining these drugs may show their synergistic impact in cardiometabolic control. This study focused on obese adults since obesity worsens dyslipidemia and hypertension and increases treatment resistance. Obese patients have insulin resistance, chronic inflammation, and endothelial dysfunction, making them appropriate for testing combined dietary and supplemental therapy. Most large-scale research on the DASH diet and omega-3 fatty acids has been done in Western cultures, whereas Asian cohorts have provided little evidence. These populations may benefit differently due to genetics, nutrition, and supplement use. Data on their uptake and applicability in obese people with hyperlipidemia and hypertension are lacking (7, 11). Therefore, the present study aimed to conduct a retrospective analysis of omega-3 fatty acid supplementation and DASH diet adherence in the management of hyperlipidemia and hypertension among obese individuals. By evaluating sociodemographic, clinical, and lifestyle characteristics in a large Chinese cohort, this study seeks to generate population-specific evidence on the role of nutritional interventions in cardiometabolic disease prevention and management.

## 2 Materials and methods

### 2.1 Study design and source of data

This retrospective study was approved by the Ethics Committee of the First Hospital of Qinhuangdao City (Approval No. 2021C017) complies with the ethical principles proposed by the Declaration of Helsinki. The study description follows the guidelines for Strengthening and Reporting in Observational Studies (STROBE).

### 2.2 Study design and setting

This cross-sectional implementation research study incorporated retrospective outcome data collection to evaluate the impact of omega-3 fatty acid supplementation and adherence to the Dietary Approaches to Stop Hypertension (DASH) diet on the management of hyperlipidemia and hypertension among obese adults. The study was undertaken at Qinhuangdao’s First Hospital from March 2023 to February 2025. To assess how nutritional intervention quality affects cardiometabolic system health, the study quantified dietary adherence, supplement use, and clinical results.

### 2.3 Participants

Participants were sequentially enrolled in the endocrinology and cardiology clinic. Participants had to be obese (28 kg/m^2^), 30–70 years old, and have hyperlipidemia and/or hypertension for 6 months. Participants were excluded from the trial due to secondary hypertension, familial hypercholesterolemia, chronic kidney disease (stage ≥3), terminal illness, and insufficient clinical and dietary data. From March 2023 to February 2025, 3,350 registered and selected after the inclusion criteria. Selectivity bias was avoided by analyzing participant demographics throughout enrollment. The sample size was sufficient to detect a 5 mmHg systolic blood pressure change or a 10 mg/dL LDL cholesterol difference between intervention and non-intervention groups with 80% power and *α* = 0.05 significance. Successful cohorts provide primary and subgroup analyses with statistical power. DASH diet adherence was assessed using a validated food frequency questionnaire. The questionnaire assessed fruit, vegetable, whole grain, low-fat dairy, lean protein, salt, and saturated fat intake. The highest tertile was “high adherence.” Prescription records, supplement purchase verification, and structured patient self-reports assessed omega-3 supplementation. Regular use was 1 g of combined EPA and DHA daily for 6 months before effect testing.

### 2.4 Fidelity and exposure assessment

Implementation adherence (degree to which patients followed DASH guidelines), dose (frequency and duration of omega-3 supplementation), quality of delivery (accuracy of dietary counseling and supplement prescription by healthcare providers), and patient responsiveness were assessed to determine fidelity of dietary interventions. Fidelity grading took into account nutritional intake, medical records, and patient adherence. Dual coding of 20% of food recollections yielded 0.87–0.93 reliability coefficients. Weighted averages of standardized domain scores yielded composite adherence scores.

### 2.5 Data collection and measures

The study measured BMI, lifestyle factors, comorbidities, medication use, and standardized lipid and blood pressure markers. Verified nutritional adherence and retrospective healthcare data enabled cross-sectional analyses.

### 2.6 Statistical analysis

All data were analyzed using standard statistical methods (SPSS version 25), using categorical variables as frequencies (%) and continuous variables as mean ± SD or median (IQR). Tests for group differences included Chi-square, ANOVA, Kruskal–Wallis, p-trend, and subgroup/multivariable regression analyses, identifying treatment interactions and independent predictors (*p* < 0.05).

## 3 Results

### 3.1 Sociodemographic, clinical, and dietary characteristics

The present study included 3,350 people with an average age of 51.8 ± 9.2 years. 52.7% of participants were female. About 31.9% of the population had completed elementary school, 38.6% had completed middle school, and 29.4% had completed high school. 32% of participants reported a moderate family income, 53% reported a low family income, and 13% reported a high income. 45.1% of married adults were married. Clinically, the average BMI was 32.8 kg/m^2^ with a standard deviation of 4.4 kg/m^2^. 46.9% had Class I obesity, 31.2% Class II, and 18.8% Class III. Seventy-one percent of patients had hypertension, with mean systolic and diastolic blood pressures of 147.2 ± 16.5 and 91.9 ± 9.6 mmHg, respectively. Hyperlipidemia was observed in 74.8% of participants, with mean total cholesterol, LDL-C, HDL-C, and triglyceride levels of 241.3 ± 40.6 mg/dL, 151.8 ± 35.7 mg/dL, 42.1 ± 8.7 mg/dL, and 211.6 ± 58.5 mg/dL, respectively. This study shows that 46.8% had a family history of cardiovascular disease, and 32.1% smoked. The DASH diet was followed by 35.5% of individuals, while 42.2% used omega-3 supplements. Also, 22.3% followed both therapies. Participants’ nutrition and treatment practices are the subject of this study. Similar to the prior study, 42.1% of the population received no treatments. The DASH diet was followed for an average of 2.2 years. However, the average period they took omega-3 supplements was 2.3 ± 1.4 years (Table 1).

**Table 1:** Sociodemographic, clinical, and dietary characteristics of participants.

Characteristic	Overall (*N* = 3,350)
Sociodemographic characteristics	
Age, mean (SD), y	51.8 (9.2)
Female (%)	1764 (52.7)
Educational attainment (%)	
– Primary school or less	1,070 (31.9)
– Middle school	1,294 (38.6)
– High school or higher	986 (29.4)
Household income (%)	
– Low	1798 (53.6)
– Middle	1,098 (32.7)
– High	454 (13.55)
Married (%)	2,518 (75.1)
Clinical characteristics	
BMI, mean (SD), kg/m^2^	32.8 (4.4)
Obesity Class, No. (%)	
– Class I (30–34.9)	1,673 (49.9)
– Class II (35–39.9)	1,048 (31.2)
– Class III (≥40)	629 (18.8)
Hypertension, No. (%)	2,403 (71.1)
Systolic BP, mean (SD), mmHg	147.2 (16.5)
Diastolic BP, mean (SD), mmHg	91.9 (9.6)
Hyperlipidemia, No. (%)	2,507 (74.8)
Total Cholesterol, mean (SD), mg/dL	241.3 (40.6)
LDL-C, mean (SD), mg/dL	151.8 (35.7)
HDL-C, mean (SD), mg/dL	42.1 (8.7)
Triglycerides, mean (SD), mg/dL	211.6 (58.5)
Family history of CVD, No. (%)	1,567 (46.8)
Smoking, No. (%)	1,077 (32.1)
Dietary and treatment characteristics	
Omega-3 supplementation (%)	1,415 (42.2)
DASH diet adherence (%)	1,189 (35.5)
Both interventions (omega-3 + DASH) (%)	746 (22.3)
No intervention, (%)	1,410 (42.1)
Duration of omega-3 use, mean (SD), y	2.3 (1.4)
Duration of DASH diet adherence, mean (SD), y	2.2 (1.3)

Data are presented as mean ± standard deviation for continuous variables and number (%) for categorical variables.

### 3.2 Dietary adherence and use of supplements

Dietary adherence and supplement use indicate how well people follow diets with nutritional assistance. In the study group (n = 3,350), 42.2% reported Omega-3 supplementation compliance, with no significant difference between males (41.2%) and females (43.1%; *p* = 0.26). Overall, the Omega-3 adherence score was 6.7 ± 1.8, with females achieving higher adherence than males (7.0 ± 1.8 vs. 6.4 ± 1.9; *p* = 0.01). The median Omega-3 use was 2.0 years (IQR 1.0–3.0) and similar between genders (*p* = 0.41). The DASH diet was adhered to by 35.5% of participants, with a larger proportion of females (37.0% vs. 33.9%; *p* < 0.001) than males. A mean DASH adherence score of 5.8 ± 2.0 was observed, with females scoring higher than males (6.0 ± 2.1 vs. 5.5 ± 1.9; *p* < 0.00). The median DASH diet adherence was 1.8 years (IQR 1.0–2.5), with no significant gender difference (*p* = 0.09). Omega-3 supplementation and the DASH diet were followed by 22.3% of individuals, with 24.9% of men and 19.9% of women (*p* = 0.004). The mean total adherence lasted 1.9 ± 1.1 years, with females having a significantly longer duration (2.1 ± 1.1 vs. 1.8 ± 1.0; *p* = 0.03) (Table 2).

**Table 2:** Dietary adherence and supplement use among participants.

Adherence category	Overall (*n* = 3,350)	Male (*n* = 1,586)	Female (*n* = 1764)	*p*-value
Omega-3 supplementation				
Compliance, No. (%)	1,415 (42.2)	654 (41.2)	761 (43.1)	0.26
Adherence score, mean (SD)^1^	6.7 (1.8)	6.4 (1.9)	7.0 (1.8)	0.01*
Duration of use, median (IQR), y	2.0 (1.0–3.0)	2.0 (1.0–3.0)	2.0 (1.0–3.0)	0.41
DASH diet adherence				
Adherence, No. (%)	1,189 (35.5)	537 (33.9)	652 (37.0)	<0.001**
Adherence score, mean (SD)^2^	5.8 (2.0)	5.5 (1.9)	6.0 (2.1)	<0.001**
Duration of adherence, median (IQR), y	1.8 (1.0–2.5)	1.7 (1.0–2.5)	1.9 (1.0–2.5)	0.09
Combined adherence (omega-3 + DASH)				
Both interventions, No. (%)	746 (22.3)	395 (24.91)	351 (19.9)	0.004**
Duration of combined adherence, mean (SD), y	1.9 (1.1)	1.8 (1.0)	2.1 (1.1)	0.03*

Data are presented as numbers (%) for categorical variables and mean ± standard deviation. Statistical comparisons between males and females were performed using chi-square tests for categorical variables and *t*-tests for continuous variables. *p* < 0.05 indicates statistical significance. ^¹^Adherence score for Omega-3 supplementation was calculated based on frequency and consistency of supplement intake. ²Adherence score for DASH diet was calculated according to dietary pattern compliance with DASH guidelines. * and ** indicate statistically significant differences (*p* < 0.05 and *p* < 0.01, respectively).

### 3.3 Lipid profile outcomes among participants

Healthy lipids reduce cardiovascular risk, while excessive levels raise heart disease and stroke risk. The combined Omega-3 supplementation and DASH diet group had the best lipid profile and blood pressure improvements at follow-up. Mean total cholesterol was lowest in the combined group (199.1 ± 30.1 mg/dL; median 199 mg/dL, IQR 180–222), compared with 211.2 ± 31.9 mg/dL in the Omega-3 only group and 207.8 ± 31.2 mg/dL in the DASH-only group (*p* < 0.001). LDL-C followed a similar pattern, with the combined group achieving the lowest levels (119.1 ± 22.6 mg/dL; median 119 mg/dL, IQR 104–137) versus 135.1 ± 25.6 mg/dL and 130.8 ± 24.9 mg/dL in the Omega-3 and DASH-only groups, respectively (*p* < 0.001). HDL-C was highest in the combined group (51.6 ± 8.7 mg/dL; median 53 mg/dL, IQR 47–59), reflecting a greater cardioprotective effect, while triglycerides were lowest (156.7 ± 44.3 mg/dL; median 155 mg/dL, IQR 135–180) compared with the other groups (*p* < 0.001 for both). The combined group had lower systolic blood pressure (136.3 ± 14.1 mmHg; median 141 mmHg, interquartile range 132–150) than the omega-3 fatty acid (141.9 ± 15.2 mmHg) and DASH (140.4 ± 14.8 mmHg) groups. The combined group had comparable diastolic blood pressure patterns, with a mean of 85.1 ± 8.2 mmHg (median of 89, interquartile range of 83–85 mmHg), compared to Omega-3 and DASH-only groups that reached 89.8 ± 9.2 and 89.1 ± 8.9 mmHg (*p* < 0.001) (Table 3).

**Table 3:** Lipid profile outcomes and target achievement among participants.

Parameter	Omega-3 Only (*n* = 1,110) mean (SD); median (IQR)	DASH Diet Only (*n* = 1,115) mean (SD); median (IQR)	Combined (*n* = 1,125) mean (SD); median (IQR)	*p*-value
Total Cholesterol (mg/dL)	211.2 (31.9); 212 (190–240)	207.8 (31.2); 204 (184–227)	199.1 (30.1); 199 (180–222)	<0.001
LDL-C (mg/dL)	135.1 (25.6); 135 (116–153)	130.8 (24.9); 128 (111–147)	119.1 (22.6); 119 (104–137)	<0.001
HDL-C (mg/dL)	47.3 (8.3); 46 (40–51)	46.9 (7.9); 48 (43–54)	51.6 (8.7); 53 (47–59)	<0.001
Triglycerides (mg/dL)	177.3 (50.4); 174 (149–200)	171.3 (47.6); 162 (139–187)	156.7 (44.3); 155 (135–180)	<0.001
Systolic BP (mmHg)	141.9 (15.2); 141 (131–151)	140.4 (14.8); 144 (134–154)	136.3 (14.1); 141 (132–150)	<0.001
Diastolic BP (mmHg)	89.8 (9.2); 93 (87–99)	89.1 (8.9); 87 (81–92)	85.1 (8.2); 89 (83–85)	<0.001

Data are presented as mean ± standard deviation and median (interquartile range) for continuous variables. Comparisons among the three intervention groups (omega-3 only, DASH diet only, and combined omega-3 + DASH) were performed using Kruskal–Wallis tests. *p* < 0.05 was considered statistically significant.

### 3.4 Anthropometric measurements among participants

Anthropometric measurements are physical assessments of body size, shape, and composition, such as height, weight, BMI, and waist circumference. At the 24-month follow-up, participants in the combined Omega-3 and DASH diet group exhibited the most significant improvements in anthropometric measures compared to those in the Omega-3 only and DASH diet only groups. The mean body weight was lowest in the combined group (83.6 ± 11.1 kg; median 81 kg, IQR 74–89), followed by the DASH diet only group (85.2 ± 12.9 kg; median 84 kg, IQR 76–92), and the Omega-3 only group (86.4 ± 12.5 kg; median 85 kg, IQR 77–93) (*p* < 0.001). Similarly, the mean BMI was lowest in the combined group (28.8 ± 3.3 kg/m^2^; median 28.8 kg/m^2^, IQR 25–30), compared to the DASH diet only group (30.1 ± 3.6 kg/m^2^; median 29.2 kg/m^2^, IQR 26–31), and the Omega-3 only group (30.5 ± 3.8 kg/m^2^; median 30.3 kg/m^2^, IQR 28–33) (*p* < 0.001). Waist circumference also showed the greatest reduction in the combined group (98.4 ± 8.7 cm; median 97 cm, IQR 91–104), followed by the DASH diet only group (101.9 ± 9.1 cm; median 101 cm, IQR 94–108), and the Omega-3 only group (104.5 ± 9.4 cm; median 103 cm, IQR 97–110) (*p* < 0.001). Notably, the combined intervention group achieved the highest proportion of participants with ≥5% weight reduction (55.5%), compared to 32.6% in the DASH diet only group and 25.6% in the Omega-3 only group (*p* < 0.001) (Table 4).

**Table 4:** Anthropometric outcomes among participants.

Anthropometric outcomes (*N* = 3,350)	Omega-3 Only (*n* = 1,110) mean (SD); median (IQR)	DASH Diet Only (*n* = 1,115) mean (SD); median (IQR)	Combined (*n* = 1,125) mean (SD); median (IQR)	*p*-value
Body weight (kg)	86.4 (12.5); 85 (77–93)	85.2 (12.9); 84 (76–92)	83.6 (11.1); 81 (74–89)	<0.001
BMI (kg/m^2^)	30.5 (3.8); 30.3 (28–33)	30.1 (3.6); 29.2 (26–31)	28.8 (3.3); 28.8 (25–30)	<0.001
Waist Circumference (cm)	104.5 (9.4); 103 (97–110)	101.9 (9.1); 101 (94–108)	98.4 (8.7); 97 (91–104)	<0.001
Achieved ≥5% weight reduction, (%)	284 (25.6)	363 (32.6)	624 (55.5)	<0.001

Data are presented as mean ± standard deviation and median (interquartile range) for continuous variables, and number (%) for categorical variables. Achieved ≥5% weight reduction refers to participants who lost at least 5% of their baseline body weight. Comparisons among the three intervention groups (omega-3 only, DASH diet only, and combined omega-3 + DASH) were performed using Kruskal–Wallis tests for continuous variables and chi-square tests for categorical variables. *p* < 0.05 was considered statistically significant.

### 3.5 Glycemic and metabolic outcomes among participants

Glycemic and metabolic outcomes refer to the effects of diet or interventions on blood sugar control, insulin response, and overall metabolic health. Participants in the combined Omega-3 supplementation and DASH diet group showed the most substantial improvements in glycemic and metabolic parameters compared with the Omega-3 only and DASH-only groups. The mean fasting glucose was lowest in the combined group (116.4 ± 19.3 mg/dL; median 115 mg/dL, IQR 103–127), compared with 126.4 ± 21.5 mg/dL in the Omega-3 only group and 123.9 ± 20.6 mg/dL in the DASH-only group (*p* < 0.001). Similarly, mean HbA1c was lowest in the combined group (6.3 ± 0.7%; median 6.3%, IQR 6.0–6.7) versus 7.1 ± 0.8% in the Omega-3 group and 6.8 ± 0.7% in the DASH-only group (*p* < 0.001). Insulin resistance, measured by HOMA-IR, was also significantly reduced in the combined group (2.3 ± 0.8; median 2.3, IQR 1.8–2.9) compared with the Omega-3 only (3.1 ± 1.0) and DASH-only groups (2.8 ± 0.9; *p* < 0.001). The combined group had the largest proportion of patients meeting target HbA1c levels, with 72.3% obtaining <7.0 and 48.4% achieving <6.5%, compared to 43.2 and 23.8% in the Omega-3 only group and 52.2 and 30.0% in the DASH-only group (p < 0.001) (Table 5).

**Table 5:** Glycemic and metabolic outcomes among participants.

Glycemic and metabolic outcomes (*n* = 3,350)	Omega-3 Only (*n* = 1,110) (mean ± SD; median [IQR])	DASH diet only (*n* = 1,115) (mean ± SD; median [IQR])	Combined (*n* = 1,125) (mean ± SD; median [IQR])	*p*-value
Fasting glucose (mg/dL)	126.4 ± 21.5; 126 [112–140]	123.9 ± 20.6; 124 [111–138]	116.4 ± 19.3; 115 [103–127]	<0.001
HbA1c, %	7.1 ± 0.8; 7.1 [6.6–7.7]	6.8 ± 0.7; 6.7 [6.3–7.2]	6.3 ± 0.7; 6.3 [6.0–6.7]	<0.001
HOMA-IR	3.1 ± 1.0; 3.0 [2.3–3.8]	2.8 ± 0.9; 2.7 [2.1–3.4]	2.3 ± 0.8; 2.3 [1.8–2.9]	<0.001
Achieved HbA1c < 7.0% (*n*, %)	479 (43.2%)	582 (52.2%)	813 (72.3%)	<0.001
Achieved HbA1c < 6.5% (*n*, %)	264 (23.8%)	334 (30.0%)	544 (48.4%)	<0.001

Data are presented as mean ± standard deviation and median (interquartile range) for continuous variables, and number (%) for categorical variables. Achieved HbA1c < 7.0 and <6.5% indicate the number and proportion of participants reaching standard glycemic control targets. Comparisons among the three intervention groups (Omega-3 only, DASH diet only, and combined omega-3 + DASH) were performed using Kruskal–Wallis tests for continuous variables and chi-square tests for categorical variables. *p* < 0.05 was considered statistically significant.

### 3.6 Combined effects of omega-3 and DASH diet on lipid and blood pressure

Participants receiving both Omega-3 supplementation and DASH diet adherence demonstrated the most pronounced improvements in lipid and blood pressure outcomes compared with either intervention alone. Mean LDL-C reduction was greatest in the combined group (−31.7 ± 9.1 mg/dL; *β* = −31.3, 95% CI –32.5 to −30.1) compared with −18.7 ± 7.3 mg/dL in the Omega-3 only group and −22.3 ± 8.1 mg/dL in the DASH-only group (*p* < 0.001; interaction *p* = 0.012). HDL-C increased most in the combined group (+5.8 ± 2.0 mg/dL; β = +5.6, 95% CI 5.3–5.9) versus +3.3 ± 1.9 mg/dL and +3.7 ± 2.0 mg/dL in the Omega-3 only and DASH-only groups, respectively (*p* < 0.001; interaction *p* = 0.021). Triglyceride reduction was also largest in the combined group (−45.3 ± 14.0 mg/dL; *β* = −44.9, 95% CI –46.7 to −43.1) compared with −28.3 ± 12.1 mg/dL and −31.7 ± 13.0 mg/dL in the Omega-3 and DASH-only groups (*p* < 0.001; interaction *p* = 0.009). Observable improvements occurred in blood pressure as well. The combined group showed the greatest reduction in systolic and diastolic BP (−14.7 ± 4.7 mmHg; β = −14.4, 95% CI –15.1 to −13.9), which is significantly greater than Omega-3 or DASH-only interventions (*p* < 0.001; interaction *p* = 0.015 and 0.022, respectively). Participants in the combined group achieved higher lipid and blood pressure targets (772/1125, 68.6%; OR = 3.74, 95% CI 3.20–4.40) in comparison to DASH-only (43.7%; OR = 1.31) and Omega-3-only (38.9%; reference), indicating a harmonious effect on cardiometabolic outcomes (p < 0.001) (Table 6).

**Table 6:** Combined effects of omega-3 and DASH diet on lipid and blood pressure.

Outcome (*n* = 3,350)	Omega-3 only (*n* = 1,110)	DASH diet only (*n* = 1,115)	Combined (*n* = 1,125)	*p*-value (Across Groups*)	Interaction effect (*p* for synergy†)
LDL-C reduction, mg/dL	−18.7 (7.3); *β* = −18.1 (95% CI: −19.2 to −17.0)	−22.3 (8.1); *β* = −22.1 (95% CI: −23.2 to −21.0)	−31.7 (9.1); *β* = −31.3 (95% CI: −32.5 to −30.1)	<0.001	0.012
HDL-C increase, mg/dL	+3.3 (1.9); *β* = +3.2 (95% CI: 3.0–3.5)	+3.7 (2.0); *β* = +3.6 (95% CI: 3.3–3.9)	+5.8 (2.0); *β* = +5.6 (95% CI: 5.3–5.9)	<0.001	0.021
Triglyceride reduction, mg/dL	−28.3 (12.1); *β* = −27.8 (95% CI: −29.5 to −26.1)	−31.7 (13.0); *β* = −31.3 (95% CI: −33.0 to −29.6)	−45.3 (14.0); *β* = −44.9 (95% CI: −46.7 to −43.1)	<0.001	0.009
Systolic BP reduction, mmHg	−7.7 (4.0); *β* = −7.5 (95% CI: −8.0 to −7.0)	−9.3 (4.2); *β* = −9.0 (95% CI: −9.5 to −8.5)	−14.7 (4.7); *β* = −14.4 (95% CI: −15.1 to −13.9)	<0.001	0.015
Diastolic BP reduction, mmHg	−4.4 (2.8); *β* = −4.3 (95% CI: −4.6 to −4.1)	−4.8 (2.9); *β* = −4.8 (95% CI: −5.1 to −4.5)	−7.7 (3.0); *β* = −7.5 (95% CI: −7.8 to −7.1)	<0.001	0.022
Achieved both lipid and BP targets, *n* (%)	432 (38.9%); OR = 1.0 (ref)	487 (43.7%); OR = 1.31 (95% CI: 1.11–1.55)	772 (68.6%); OR = 3.74 (95% CI: 3.20–4.40)	<0.001	**—**

Data are presented as mean ± standard deviation for continuous outcomes, with regression β coefficients and 95% confidence intervals (CI), and number (%) with odds ratios (OR) and 95% CI for categorical outcomes. Comparisons among the three intervention groups (Omega-3 only, DASH diet only, and combined omega-3 + DASH) were performed using analysis of covariance (ANCOVA) for continuous variables and logistic regression for categorical variables. Interaction effects (p for synergy) indicate whether the combined intervention produced effects greater than the sum of individual interventions. *p* < 0.05 was considered statistically significant. *p-value indicates statistical comparison across the three intervention groups (Omega-3 only, DASH diet only, and combined). ^†^*p* for synergy represents the interaction effect, showing whether the combined intervention produced effects greater than the sum of the individual effects.

### 3.7 Subgroup analyses of combined omega-3 and DASH diet effects on lipid and blood pressure

Omega-3 supplementation and DASH diet intervention reduced LDL-C, systolic blood pressure, and met both targets in all categories, with considerable variation in degree. Participants aged ≥60 years experienced slightly greater LDL-C reduction (−31.2 ± 9.3 mg/dL; *β* = −30.6, 95% CI –31.7 to −29.5) and systolic BP reduction (−15.4 ± 4.8 mmHg; *β* = −15.1, 95% CI –15.8 to −14.4) compared with those <60 years, and a higher proportion achieved both targets (68.8% vs. 66.1%; OR = 3.90 vs. 3.44; interaction *p* = 0.036). Gender-based analysis showed slightly greater improvements among females, with LDL-C reduction of −31.6 ± 9.1 mg/dL (*β* = −31.2, 95% CI –32.3 to −30.1) and systolic BP reduction of −14.7 ± 4.7 mmHg (*β* = −14.6, 95% CI –15.3 to −13.9), resulting in 68.2% achieving both targets (OR = 3.76; interaction *p* = 0.041). Duration of obesity influenced outcomes, with participants obese ≥5 years demonstrating larger LDL-C reductions (−32.2 ± 9.4 mg/dL; *β* = −32.1, 95% CI –33.2 to −31.0) and systolic BP decreases (−15.3 ± 4.8 mmHg; β = −15.2, 95% CI –15.9 to −14.5) compared with those obese <5 years, and a higher proportion achieving both targets (68.8% vs. 66.2%; interaction *p* = 0.025). Diabetes status was a strong modifier of intervention effectiveness: participants with diabetes achieved the greatest LDL-C reduction (−33.4 ± 9.5 mg/dL; β = −33.2, 95% CI –34.4 to −32.1), systolic BP reduction (−15.3 ± 5.0 mmHg; β = −15.2, 95% CI –15.9 to −14.5), and proportion achieving both targets (70.6%; OR = 4.26) compared with non-diabetic participants (64.8%; OR = 3.20; interaction *p* = 0.01) (Table 7).

**Table 7:** Subgroup analyses of combined omega-3 and DASH diet effects on lipid and blood pressure.

Subgroup (*n* = 3,350)	LDL-C Reduction, mg/dL (Mean ± SD; β [95% CI])	Systolic BP reduction, mmHg (Mean ± SD; β [95% CI])	Achieved both targets, % (OR [95% CI])	*p*-value (Subgroup × Treatment interaction)
Age				
<60 years (*n* = 1955)	−29.5 (9.2); *β* = −29.3 (−30.4 to −28.2)	−13.1 (4.4); *β* = −13.1 (−13.7 to −12.5)	66.1%; OR = 3.44 (2.87–4.13)	0.036
≥60 years (*n* = 1,395)	−31.2 (9.3); *β* = −30.6 (−31.7 to −29.5)	−15.4 (4.8); *β* = −15.1 (−15.8 to −14.4)	68.8%; OR = 3.90 (3.30–4.61)	
Gender				
Male (*n* = 1,586)	−30.6 (9.2); *β* = −30.2 (−31.3 to −29.1)	−14.4 (4.6); *β* = −14.1 (−14.8 to −13.4)	67.4%; OR = 3.68 (3.10–4.37)	0.041
Female (*n* = 1764)	−31.6 (9.1); *β* = −31.2 (−32.3 to −30.1)	−14.7 (4.7); *β* = −14.6 (−15.3 to −13.9)	68.2%; OR = 3.76 (3.16–4.47)	
Duration of obesity				
<5 years (*n* = 1,410)	−28.5 (868); *β* = −28.2 (−29.3 to −27.1)	−13.9 (4.4); *β* = −13.4 (−14.0 to −12.8)	66.2%; OR = 3.34 (2.77–4.03)	0.025
≥5 years (*n* = 1940)	−32.2 (9.4); *β* = −32.1 (−33.2 to −31.0)	−15.3 (4.8); *β* = −15.2 (−15.9 to −14.5)	68.8%; OR = 3.94 (3.34–4.65)	
Diabetes status				
With diabetes (*n* = 1780)	−33.4 (9.5); *β* = −33.2 (−34.4 to −32.1)	−15.3 (5.0); *β* = −15.2 (−15.9 to −14.5)	70.6%; OR = 4.26 (3.62–5.02)	0.01
Without diabetes (*n* = 1,570)	−28.7 (8.8); *β* = −28.5 (−29.6 to −27.4)	−13.9 (4.5); *β* = −13.7 (−14.2 to −13.1)	64.8%; OR = 3.20 (2.68–3.81)	—

Data are presented as mean ± standard deviation for continuous outcomes, with regression β coefficients and 95% confidence intervals (CI), and number (percentage) with odds ratios (OR) and 95% CI for categorical outcomes. Subgroups analyzed include age (<60 vs. ≥60 years), gender (male vs. female), duration of obesity (<5 vs. ≥5 years), and diabetes status (with vs. without diabetes). Subgroup × treatment interaction *p*-values indicate whether the effect of the combined intervention differs significantly across subgroups. Comparisons were performed using regression models for continuous outcomes and logistic regression for categorical outcomes. *p* < 0.05 was considered statistically significant.

### 3.8 Time-dependent trends in lipid, blood pressure, and weight outcomes by intervention

All intervention groups showed improvement in weight, blood pressure, and lipids at 24 months interval. Total cholesterol steadily decreased, with the Omega-3 + DASH group showing the greatest reduction from baseline (239.4 ± 32.1 mg/dL) to 24 months (201.5 ± 25.2 mg/dL). The combined group’s LDL-C reduced from 148.8 ± 24.7 mg/dL at baseline to 113.9 ± 20.1 at 24 months. HDL-C levels increased in all groups, especially the combination group (from 38.7 ± 8.3 to 44.6 ± 8.0 mg/dL). The combo intervention was the most effective in lowering triglycerides (from 186.5 ± 43.1 to 153.9 ± 36.1 mg/dL). The combined group had the greatest decrease in systolic and diastolic blood pressures over time (systolic BP: 148.4 ± 12.7 mmHg at baseline to 131.2 ± 9.4 at 24 months; diastolic BP: 92.5 ± 82.6 ± 7.1 mmHg). The combined group experienced the most weight decrease (≥5%) at 24 months (46.6%). 58.7% met cholesterol and blood pressure objectives at 24 months. Combining Omega-3 supplementation with the DASH diet led to sustained cardiometabolic advantages (*p*-trend < 0.001) (Table 8).

**Table 8:** Time-dependent trends in lipid, blood pressure, and weight outcomes by intervention group.

Outcome (*n* = 3,350)	Time	Omega-3 only (*n* = 1,110)	DASH diet only (*n* = 1,115)	Combined (*n* = 1,125)	p-trend (Across groups)
Total cholesterol, mg/dL	Baseline	238.8 ± 31.7	240.2 ± 32.3	239.4 ± 32.1	<0.001
	6 months	215.8 ± 29.8	212.3 ± 29.4	210.8 ± 27.6	
	12 months	209.3 ± 27.2	204.8 ± 26.1	202.8 ± 25.1	
	24 months	205.9 ± 26.9	202.8 ± 25.7	201.5 ± 25.2	
LDL-C, mg/dL	Baseline	147.6 ± 24.0	148.7 ± 25.1	148.8 ± 24.7	<0.001
	6 months	125.9 ± 22.1	123.1 ± 21.3	121.1 ± 21.0	
	12 months	120.5 ± 20.9	117.6 ± 19.9	115.2 ± 19.9	
	24 months	118.8 ± 20.6	115.6 ± 19.7	113.9 ± 20.1	
HDL-C, mg/dL	Baseline	38.5 ± 8.2	39.2 ± 8.6	38.7 ± 8.3	0.021
	6 months	41.8 ± 8.0	42.6 ± 7.8	42.6 ± 8.1	
	12 months	42.5 ± 8.4	43.6 ± 8.3	43.9 ± 8.2	
	24 months	43.1 ± 8.1	44.0 ± 8.2	44.6 ± 8.0	
Triglycerides, mg/dL	Baseline	183.1c ± 41.7	185.3 ± 42.2	186.5 ± 43.1	<0.001
	6 months	161.4 ± 38.4	161.5 ± 39.2	158.9 ± 37.9	
	12 months	159.8 ± 36.9	158.6 ± 37.1	156.4 ± 36.4	
	24 months	157.2 ± 36.4	155.8 ± 36.8	153.9 ± 36.1	
Systolic BP, mmHg	Baseline	147.9 ± 12.4	148.6 ± 12.6	148.4 ± 12.7	<0.001
	6 months	138.6 ± 10.0	135.7 ± 10.2	135.2 ± 10.1	
	12 months	134.5 ± 9.7	132.8 ± 9.6	131.4 ± 9.7	
	24 months	133.8 ± 9.7	131.6 ± 9.5	131.2 ± 9.4	
Diastolic BP, mmHg	Baseline	91.7 ± 8.3	92.3 ± 8.6	92.5 ± 8.5	<0.001
	6 months	85.4 ± 7.4	85.7 ± 7.7	85.4 ± 7.4	
	12 months	83.6 ± 7.0	84.2 ± 7.6	83.9 ± 7.3	
	24 months	82.8 ± 7.0	83.1 ± 7.2	82.6 ± 7.1	
≥5% Weight loss, No. (%)	6 months	254 (22.9)	296 (26.5)	318 (28.3)	<0.001
	12 months	460 (41.4)	489 (43.9)	495 (44.0)	<0.001
	24 months	482 (43.4)	513 (46.0)	524 (46.6)	
Achieved lipid and BP targets, No. (%)	6 months	377 (34.0)	385 (34.5)	388 (34.05)	<0.001
	12 months	601 (54.1)	616 (55.2)	620 (55.1)	
	24 months	628 (56.6)	644 (57.8)	660 (58.7)	

Data are presented as mean ± standard deviation for continuous variables and number (%) for categorical variables. Measurements were taken at baseline, 6 months, 12 months, and 24 months for each intervention group (Omega-3 only, DASH diet only, and combined omega-3 + DASH). p-trend (Across Groups) indicates the overall statistical significance of changes over time among the three groups. *p* < 0.05 was considered statistically significant.

### 3.9 Adverse events and safety outcomes in omega-3 and DASH diet interventions

Adverse events and safety outcomes evaluate the risks, side effects, and overall tolerability of omega-3 and DASH diet interventions. Table 9 summarizes adverse events and safety outcomes for participants receiving omega-3 supplementation, the DASH diet, or a combination of both interventions. Gastrointestinal upset was reported in 12.6% of the omega-3 group, 8.1% of the DASH diet group, and 15.6% of the combined group, showing a statistically significant difference (*p* = 0.017). Fishy aftertaste or reflux occurred in 17.4% of the omega-3 group and 18.7% of the combined group, with no data available for the DASH diet group, and this difference was highly significant (*p* < 0.001). Minor bleeding events were observed in 5.2% of the omega-3 group, 1.9% of the DASH diet group, and 6.2% of the combined group, with a significant difference across groups (*p* = 0.008). Hypotension-related symptoms were reported in 5.5% of the omega-3 group, 6.7% of the DASH diet group, and 9.0% of the combined group (*p* = 0.046). Poor diet tolerability or withdrawal due to DASH diet restrictions was 11.7% in the DASH diet group and 8.3% in the combined group, while not applicable to the omega-3 group, with a significant difference (*p* = 0.032). Hospitalization, gastrointestinal bleeding, and cardiovascular events were rare: 0.8% in the omega-3 group, 0.5% in the DASH diet group, and 1.2% in the combination group (*p* = 0.419). Omega-3 had 87.2% adherence, DASH 83.6%, and the combined group 81.4% despite adverse effects.

**Table 9:** Adverse events and safety outcomes in omega-3 and DASH diet interventions (*N* = 3,350).

Adverse event/safety parameter	Omega-3 only (*n* = 1,110)	DASH diet only (*n* = 1,115)	Combined (*n* = 1,125)	*p*-value
Gastrointestinal upset, No. (%)	141 (12.6)	89.4 (8.1)	174 (15.6)	0.017
Fishy aftertaste/reflux, No. (%)	194 (17.4)	–	209 (18.7)	<0.001
Minor bleeding events (epistaxis, bruising), No. (%)	59 (5.2)	21.2 (1.9)	68.3 (6.2)	0.008
Hypotension-related symptoms (dizziness, lightheadedness), No. (%)	62 (5.5)	75 (6.7)	101 (9.0)	0.046
Poor diet tolerability/withdrawal due to DASH restrictions, No. (%)	–	133 (11.7)	95 (8.3)	0.032
Serious adverse events (hospitalization, GI bleed, CV event), No. (%)	8.4 (0.8)	5.2 (0.5)	11.5 (1.2)	0.419
Overall adherence despite side effects, %	87.2	83.6	81.4	

Categorical variables are presented as counts and percentages. Between-group comparisons were conducted using chi-square tests. *p*-values <0.05 were considered statistically significant. Overall adherence remained high across all groups (>80%) despite the occurrence of mild-to-moderate adverse events. Missing data, if any (<5%), were handled using appropriate imputation methods. A significance threshold of *p* < 0.05 was applied.

### 3.10 Multivariable regression analysis of lipid and blood pressure

Multivariable regression showed that adherence considerably affects outcomes. High omega-3 supplementation adherence (≥80%) significantly enhanced odds of attaining cholesterol and blood pressure targets (OR: 1.81, 95% CI: 1.40–2.33, *p* < 0.001), while high DASH diet adherence alone had even greater odds (OR: 2.16, 95% CI: 1.68–2.77, *p* < 0.001) Adherence to both medications resulted in the best success rate (OR: 3.03, 95% CI: 2.32–3.96, p < 0.001). Among baseline characteristics, participants with BMI < 30 kg/m^2^ (*β* = −0.26, SE = 0.10; OR: 1.32, 95% CI: 1.07–1.63, *p* = 0.004), obesity duration <5 years (OR: 1.22, 95% CI: 1.02–1.44, *p* = 0.038), and a Charlson comorbidity index <2 (OR: 1.40, 95% CI: 1.16–1.69, p < 0.001) showed significantly greater odds of achieving targets. In contrast, female sex (OR: 1.11, 95% CI: 0.94–1.29, *p* = 0.312), younger age <60 years (OR: 1.19, 95% CI: 0.97–1.47, *p* = 0.11), and baseline hypertension (OR: 0.86, 95% CI: 0.72–1.04, *p* = 0.176) were not significant predictors. Diabetes was found to be negatively linked with outcomes (OR: 0.74, 95% CI: 0.63–0.89, p < 0.001), indicating a lower likelihood of achieving cholesterol and BP control (Table 10).

**Table 10:** Multivariable regression analysis of predictors of lipid and blood pressure control.

Predictor variable (*N* = 3,350)	*β* coefficient (SE)	Adjusted OR (95% CI)	*p*-value
High adherence to omega-3 supplementation (≥80%)	–	1.81 (1.40–2.33)	<0.001
High adherence to DASH guidelines (≥80%)	–	2.16 (1.68–2.77)	<0.001
Combined adherence (Omega-3 + DASH ≥80%)	–	3.03 (2.32–3.96)	<0.001
Baseline BMI < 30 kg/m^2^	−0.26 (0.10)	1.32 (1.07–1.63)	0.004
Duration of obesity <5 years	–	1.22 (1.02–1.44)	0.038
Female sex	–	1.11 (0.94–1.29)	0.312
Age <60 years	–	1.19 (0.97–1.47)	0.11
Presence of diabetes (Yes vs. No)	–	0.74 (0.63–0.89)	<0.001
Hypertension at baseline (Yes vs. No)	–	0.86 (0.72–1.04)	0.176
Charlson comorbidity index <2	–	1.4- (1.16–1.69)	<0.001

Logistic regression was applied since the outcome (achievement of lipid and blood pressure targets) is binary. *β* coefficients (with SE) describe the direction and strength of associations on the log-odds scale. Adjusted odds ratios (ORs) with 95% CIs show the relative likelihood of achieving targets while controlling for confounders. *p*-values assess statistical significance, with values <0.05 considered significant.

## 4 Discussion

This study investigates the comparative and combined effects of Omega-3 fatty acids and the DASH diet on lipid profile and blood pressure regulation in obese individuals with hyperlipidemia and hypertension. According to previous studies, obesity is strongly linked to hypertension and dyslipidemia (11, 12). The Dietary Approaches to Stop Hypertension (DASH) trial found that diet changes help to lower blood pressure (13, 14). Many patients did not follow DASH guidelines, showing an implementation gap similar to those in other Asian nations where cultural eating patterns and minimum nutritional guidance are not widely accepted (15). Our study showed that people are more adherent to omega-3 supplements than the DASH diet, which is consistent with international trends in triglyceride lowering (16). High-risk patients may benefit from dose-dependent Omega-3 fatty acid triglyceride and cardiovascular event reduction. Due to our mean triglyceride level of 212 mg/dL, omega-3 intervention is necessary. VLDL, triglycerides, inflammation, and atherosclerotic plaques decrease in the liver (17, 18). Limiting sodium, increasing potassium, magnesium, and fiber, and reducing saturated fat may improve endothelial function, insulin sensitivity, and vascular tone, according to previous studies (7, 19). A subset of participants using DASH and omega-3 simultaneously may have caused the combined group’s synergistic improvements in lipid and blood pressure outcomes, supporting controlled studies that show multi-targeted lifestyle and nutraceutical interventions work better than isolated interventions (12, 20). Recently, a national survey in China demonstrated that obesity prevalence has consistently increased over the previous decade (4). This study indicated that our community has high hypertension and hyperlipidemia and low evidence-based diet adherence, suggesting public health programs should improve. Practical adherence and obesity-related cardiovascular disease combination methods are stressed. This study proves DASH and omega-3 therapy’s cardiometabolic benefits. According to US and European data, women had greater diet quality and cardioprotective diet engagement than men (21). For instance, analysis from the Nurses’ Health Study and Health Professionals Follow-Up Study indicated that women were more likely to sustain adherence to DASH and Mediterranean-style diets over long-term follow-up (22). Asian women are more likely to use omega-3 supplements for weight and cardiovascular risk. Our omega-3 compliance rate was 42.2%, compared to 20–35% in Western trials. Our findings are consistent with urban Chinese groups with high nutraceutical therapy awareness (23–25). Similar to Chinese data, DASH diet acceptance has remained below 40% despite its proven benefits in lowering blood pressure and improving cardiometabolic outcomes (26). The low total adherence rate (22.3%) suggests that multiple concurrent dietary treatments are difficult to apply, validating recent studies that suggest dietary complexity may affect compliance in real life (27). Furthermore, females may have longer combined adherence as they are more health-conscious and adopt proactive preventative approaches (28). These findings show that obese people may need gender-specific interventions to increase long-term omega-3 supplementation and DASH dietary recommendations.

It is generally known that omega-3 fatty acids decrease triglycerides and enhance HDL-C. Meta-analyses suggest a 20–30% decline in triglycerides and a 5–10% increase in HDL-C (18, 29). Similarly, the DASH diet has also been associated with reductions in total cholesterol and LDL-C, as demonstrated in the PREMIER and OmniHeart trials, which reported LDL-C reductions of 10–15 mg/dL over 6 months (26, 30). Omega-3 and DASH may cooperate. Omega-3 s target triglyceride-rich lipoproteins, while DASH reduces saturated fat, salt, LDL-C, and total lipids. Recent observational and interventional research support multi-target diets. Recent studies found that fish-based and DASH diets regulated lipids better than either alone (31). A meta-analysis found that multi-component diets improved cardiometabolic outcomes over single-therapy (32). Previous researches showed that obese people with hyperlipidemia and hypertension benefit from a combined strategy (14, 20, 33, 34). Recent studies found that multi-component diet and lifestyle treatments could lower SBP by 10 mmHg, improving hypertension management (7, 14). Omega-3 supplementation with DASH diet adherence improved weight, BMI, and waist circumference more than either alone. Research shows DASH fruits, veggies, healthy grains, and low-fat dairy prevent belly obesity and small weight loss. Omega-3 s lower inflammation, regulate lipids, and improve insulin sensitivity (26, 35). The combined intervention may result in ≥5% weight loss due to synergistic effects such enhanced satiety, reduced calorie intake, and improved metabolic regulation. Omega-3 supplementation with the DASH diet permanently reduces weight and improves central adiposity in overweight and obese persons.

The DASH diet also improves glycemic control and insulin sensitivity in patients with metabolic syndrome. According to randomized controlled studies and meta-analyses, the DASH diet decreases fasting glucose and HbA1c by emphasizing high fiber, low sodium, and reduced refined carbs (36, 37). Omega-3 fatty acids reduce inflammation and increase cell membrane fluidity, which may stimulate insulin signaling, while the DASH diet lowers food glycemic load to optimize postprandial glucose levels. A recent study found that Mediterranean-style diets with omega-3-rich foods improved glycemic results more than either alone (38). A systematic study found that DASH diet adherence lowered HbA1c and insulin resistance, especially when paired with weight loss and nutrient-specific supplementation (39). The current study found that omega-3 fatty acid supplementation with the DASH diet improves glycemic and metabolic outcomes more than either alone. This comprehensive metabolic control and cardiovascular risk reduction approach may assist type 2 diabetes and prediabetes patients. Omega-3 fatty acids and the DASH diet enhance cardiometabolic risk factors individually and synergistically, according to this study. Hypertensive and metabolic syndrome patients’ cholesterol and blood pressure drop on the DASH diet (22, 35, 40). Meanwhile, omega-3 fatty acids are well documented to reduce triglycerides and modestly increase HDL-C, though their effect on LDL-C and blood pressure is less robust (7, 13, 33). Synergistic benefits of DASH, omega-3 s, and mixed diets, such as Mediterranean diets with omega-3, improve lipid indicators and endothelial function (7, 16, 20, 38, 41). The REDUCE-IT trial found that omega-3 fatty acid therapy improved triglyceride reduction and cardiovascular outcomes (42, 43). A study showed weight loss in the first year, partial weight rebound, and weak dietary and supplement adherence (35). This study shows that omega-3 supplementation within a DASH framework may be the optimal nutritional therapy for cardiovascular disease patients, delivering clinically significant benefits. Despite strong adherence, omega-3 supplementation caused gastrointestinal discomfort (44), fishy taste, and slight bleeding. The DASH diet lowers blood pressure, which may worsen hypotension. The strict diet raised withdrawal rates, emphasizing long-term adherence issues (14, 45).

This study validates prior findings that adherence improves cardiometabolic health. The DASH and PREMIER studies found that diet compliance reduced blood pressure and cholesterol more (46). Similarly, meta-analyses of omega-3 supplementation show that dose and adherence strongly influence lipid and blood pressure outcomes (47, 48). The synergistic advantage of omega-3 and DASH here matches evidence showing multi-component lifestyle therapies improve cardiovascular risk variables more than single programs (7, 36). Diabetics responded less, corroborating prior results that severe metabolic dysfunction degrades lifestyle-only therapy and typically requires pharmacological co-management (49, 50). These data underline the need of adherence and early intervention in improving cardiometabolic outcomes with the Dash diet and Omega-3 supplementation.

## 5 Conclusion

DASH diet and omega-3 fatty acid supplementation improve lipid metabolism, blood pressure regulation, glycemic control, and anthropometrics among obese adults having hyperlipidemia and hypertension. Omega-3 fatty acids with the DASH diet may reduce hepatic triglyceride synthesis, increase *β*-oxidation, and minimize vascular inflammation. The DASH diet is enriched with fiber and antioxidants, which improve endothelial function, vasodilation, and insulin sensitivity. Combining interventions increased the likelihood of meeting dual cholesterol and blood pressure targets three times, highlighting its therapeutic importance for cardiometabolic risk reduction. Subgroup studies show that older adults, women, people with long-term obesity, and diabetics are more affected, suggesting precision nutrition interventions may be stratified. Prospective, multicenter, randomized trials are needed to demonstrate causality and identify omega-3 supplement content, dosage, and duration in conjunction with DASH adherence. Synergistic effects will be explained by mechanistic studies using biomarkers of inflammation, endothelial function, and lipid kinetics. Digital health tools, behavioral interventions, and culturally specific education may improve long-term adherence and clinical effectiveness. Expanding the study to other Asian communities with different genetic and dietary origins can help produce population-specific, evidence-based nutritional advice to reduce obesity-related cardiovascular disease.

## Data Availability

The original contributions presented in the study are included in the article/supplementary material, further inquiries can be directed to the corresponding author/s.
